# Estimation of Serum 1,25-Dihydroxycholecalciferol and Tumor Necrosis Factor-α Levels in Chronic Periodontitis

**DOI:** 10.7759/cureus.45896

**Published:** 2023-09-25

**Authors:** Ravindranath Dhulipalla, CH L Sowjanya, Lakshmikanth Kolaparthy, Ramanarayana Boyapati, Chaitanya Adurty, Yamuna Marella

**Affiliations:** 1 Periodontology, Sibar Institute of Dental Sciences, Guntur, IND

**Keywords:** 1 25 dihydroxy cholecalciferol, elisa, tumor necrosis factor alpha, pro inflammatory cytokines, chronic periodontitis

## Abstract

Background: Major trials in the field of periodontics include a thorough understanding of its pathophysiology and the interplay between host response and periodontal factors. Certain factors such as vitamin D play a crucial role in immune regulation and their depletion is known to contribute to the onset of periodontitis. Research efforts continue to unravel the impact of elevated pro-inflammatory cytokines like TNF-α on osteoclastogenesis in periodontitis.

Materials and methods: The study comprised a total of 64 participants, with 33 men and 31 women, including 32 individuals with chronic periodontitis and 32 people without the condition. ELISA was employed to determine the concentrations of TNF-α and 1,25-dihydroxycholecalciferol (1,25(OH)_2_D) in the participants. Clinical attachment levels, probing pocket depth, gingival bleeding index, and plaque index were meticulously measured. Subsequent to data collection, appropriate statistical tests were conducted.

Results: The mean serum levels of 1,25(OH)_2_D in test and control groups are 173.59 ± 52.60 and 401.47 ± 99.81, respectively. The mean serum levels of TNF-α in the test and control groups are 1078.09 ± 231.51 and 204.75 ± 68.31, respectively. The TNF-α levels exhibited a statistically significant difference between test and control groups (*p* = 0.0001) at a 5% level of significance.

Conclusion: Decreased levels of 1,25(OH)_2_D led to increased values of periodontal parameters. There was also a significant increase in serum levels of pro-inflammatory cytokines such as TNF-α.

## Introduction

Chronic periodontitis is a multifactorial infectious disease that affects the supporting tissues and is characterized by progressive attachment loss and bone degradation, ultimately leading to tooth loss. The exact mechanism of chronic periodontitis is still not fully understood. However, one of the possible causes is the interaction between host and microbial factors. These interactions can trigger the release of inflammatory molecules, such as interleukins and tumor necrosis factor (TNF), which can lead to alveolar bone loss and periodontitis [1]. Additional risk factors include plaque microbes, poor oral hygiene, socio-behavioral variables such as smoking, hereditary and systemic conditions such as diabetes and osteoarthritis, and tooth-level factors such as abnormal tooth position and frenal attachment [2].

Gene polymorphisms affecting vitamin D receptors exert a significant influence on bone and mineral metabolism, innate immunity, and other processes. Furthermore, these polymorphisms have been linked to gum disease. The anti-inflammatory and immune-modulating properties of vitamin D also play a role in the maintenance of good dental health [3]. However, a substantial intrigue persists surrounding the impact of vitamin D levels on periodontal health. Existing evidence suggests a correlation between severe periodontitis and low vitamin D levels. Vitamin D deficiency has been associated with increased bleeding on probing (BOP) and a decline in periodontal attachment compared to individuals with adequate vitamin D levels. In animal models, vitamin D has demonstrated the capability to diminish the number of Porphyromonas gingivalis and mitigate the inflammatory burden of periodontitis [3].

Both immune apparatus cells and epithelial cells are equipped to express the enzyme 1α-hydroxylase also known as CYP27B1 and synthesize calcitriol, which facilitates the vitamin D autocrine pathway [4]. An active biological metabolite of vitamin D, 1,25-dihydroxcholecalciferol (1,25(OH)2D), binds to the vitamin D receptor, consequently engaging with a responsive gene such as one encoding a calcium-binding protein. This interaction serves as an indicator of an individual’s vitamin D status. Possible mechanisms through which vitamin D3 exerts its effects include the activation of mitogen-activated protein kinases and nuclear factor-kappa B [5]. TNF-α is produced by monocytes and/or macrophages, while TNF-β is synthesized by lymphoid cells. The latter is concerned with signaling pathways involving NF-kB/p65. They also downregulate the mRNA expression levels, and also at high levels along with IL-1, release vasoactive amines such as serotonin, histamine, platelet-activating factor (PAF) as well as prostaglandins [6]. In the matter of immunity regulation and osteoclastogenesis via the RANKL-OPG axis, vitamin D and TNF-α are pivotal in the pathogenetic analysis of bone-loss disorders associated with inflammation, including periodontitis [7]. Studies reveal that vitamin D can modulate cytokine production through diverse mechanisms, thereby mitigating TNF-α’s propensity for bone resorption [8,9]. Although bone resorption is triggered by vitamin D in the initial stages, prolonged exposure to vitamin D promotes osteoblastic growth [10].

This research aims to quantify 1,25(OH)2D and TNF levels in the bloodstreams of individuals afflicted with chronic periodontitis and those with healthy gums. Serum 1,25(OH)2D and TNF values were compared between patients suffering from chronic periodontitis and healthy volunteers for the first time in this investigation.

## Materials and methods

Study design

This study was structured as a cross-sectional analysis, involving a comparison of serum vitamin D and TNF-α levels in chronic periodontitis patients and periodontally healthy individuals.

Participants

The participant pool consisted of 64 systemically healthy patients; those without medical conditions aged ≥35 years, with ≥15 teeth present, displaying tooth mobility ≤grade I, and exhibiting full mouth plaque scores (FMPS) <20% at baseline. Participants were selected from the outpatient department of periodontics based on their willingness.

The exclusion criteria were pregnant or breastfeeding parents, smokers, periodontal surgery within one year, or those who displayed signs of untreated acute infection at the surgical site, exhibited apical pathology or severe root irregularities, or had received antibiotic therapy in the preceding six months.

Prior to participation, patients provided their written informed consent. The research unfolded over a 12-month period and received ethical approval from the institutional ethical committee. The test group comprised patients with generalized chronic periodontitis, while the control group comprised periodontally healthy subjects. The diagnostic framework aligned with the criteria established by the AAP classification of periodontal disease in 1999 [11]. According to this classification, patients with >30% of sites with probing depths ≥5 mm and clinical attachment loss ≥4 mm were included in the generalized chronic periodontitis group. 

The IRB of our institution approved the study with the clearance number Pr.10/IEC/SIBAR/2015. 

Procedure

Firstly, five milliliters of blood was aseptically drawn from the antecubital fossa using a sterile syringe to maintain a germ-free environment. Hematological parameters, including serum 1,25(OH)2D and serum TNF-α levels, were assessed from the collected blood samples using enzyme-linked immunosorbent assay (ELISA). Secondly, the estimation of human 1,25(OH)2D was conducted through an ELISA kit employing a biotin double antibody sandwich technique. The resulting hue of the solution exhibited a discernible correlation with the concentration of human 1,25(OH)2D. Then, the levels of TNF-α were determined using an in vitro ELISA kit developed by RayBiotech (RayBiotech Life Inc., Peachtree Corners, Georgia). This kit is designed for the determination of human TNF-α in blood, plasma, and cell culture supernatants. The strength of the color shift from blue to yellow, as measured at 450 nm, serves as a reliable indicator of TNF-α concentration.

To examine the periodontal state, a UNC-15 periodontal probe was systematically utilized to evaluate six specific locations. Parameters including the Silness-Loe Plaque Index, Gingival Bleeding Index, probing pocket depth, and clinical attachment level were recorded.

Statistical analysis

The statistical analysis was performed using SPSS 20.0 (IBM Corp., Armonk, NY). Group comparisons were conducted using the Mann-Whitney U test and the t-test was used to compare 1,25(OH)2D and TNF-α levels in the blood. The relationship between periodontal and hematological parameters within the experimental and control groups was analyzed using Karl Pearson's correlation coefficient. The 95% CI was calculated and the key characteristics were identified. The p-value of 0.05 was deemed statistically significant.

## Results

This study comprised a total of 64 participants (32 with chronic periodontitis and 32 periodontally healthy individuals). Out of these participants, 33 were male, and 31 were female, with a mean age of 42.91 years (Table 1).

**Table 1:** Distribution of patients in control and test groups according to age

Age group	Control group	%	Test group	%	Total	%
30-39yrs	11	34.37	12	37.50	23	35.94
40-49yrs	16	50.00	16	50.00	32	50.00
50+yrs	5	15.63	4	12.50	9	14.06
Total	32	100.00	32	100.00	64	100.00
Mean age	42.16		43.66		42.91	
Standard Deviation age	6.35		9.23		7.89	

The distribution of various parameters in the control and test groups followed a normal pattern (Table 2).

**Table 2:** Normality of different parameters in control and test groups by Kolmogorov Smirnov test p value less than 0.05 is statistically significant

Time points	Control group		Test group	
	Z-value	P-value	Z-value	P-value
Plaque index	0.56	0.91	0.62	0.83
Gingival bleeding index	0.48	0.97	0.80	0.53
Pocket depth	0.57	0.89	0.66	0.76
Clinical attachment level	0.63	0.81	0.47	0.97
Serum TNF-alpha levels	0.97	0.30	0.49	0.96
Serum tumor necrosis factor-alpha levels	0.88	0.41	1.08	0.13

Analysis of periodontal parameters

In the test group, the mean plaque index score is 1.49 ± 0.29, whereas in the control group, it is 0.42 ± 0.13. Notably, a statistically significant difference was observed between the test and control groups in terms of plaque index (p = 0.0001) (Table 3), gingival bleeding index (p = 0.0001) (Table 4), probing pocket depth, and clinical attachment level with a p = 0.0001 (Tables 5-6).

**Table 3:** Comparison of control and test groups with respect to plaque index scores SD: standard deviation p value less than 0.05 is statistically significant

Groups	Mean	SD	P-value
Control group	0.42	0.13	
Test group	1.49	0.29	0.0001

**Table 4:** Comparison of control and test groups with respect to gingival bleeding index scores by independent test SD: standard deviation SE: standard error p value less than 0.05 is statistically significant

Groups	Mean	SD	SE	t-value	P-value
Control group	23.84	8.89	1.57		
Test group	84.73	9.76	1.73	-26.08	0.0001

**Table 5:** Comparison of control and test groups with respect to pocket depth scores by independent t-test p value less than 0.05 is statistically significant SD: standard deviation SE: standard error

Groups	Mean	SD	SE	t-value	P-value
Control group	1.80	0.28	0.05		
Test group	5.25	0.57	0.10	-30.72	0.0001

**Table 6:** Comparison of control and test groups with respect to clinical attachment level scores by independent t-test p value less than 0.05 is statistically significant SD: standard deviation SE: standard error

Groups	Mean	SD	SE	t-value	P-value
Control group	1.64	0.21	0.04		
Test group	5.68	0.57	0.10	-37.66	0.0001

Correlation analysis among periodontal parameters in both test and control groups (Table 7) showed that the plaque index shared a statistically significant correlation with both probing depth and clinical attachment level.

**Table 7:** Correlation among periodontal parameters in test and test groups by Karl Pearson’s correlation coefficient method p value less than 0.05 is statistically significant SD: standard deviation SE: standard error

Periodontal parameters	Control group				Test group			
	Plaque index	Gingival bleeding index	Pocket depth	Clinical attachment level	Plaque index	Gingival bleeding index	Pocket depth	Clinical attachment level
Plaque index	-				-			
Gingival bleeding index	r=0.13	-			r =0.27	-		
Pocket depth	r=0.37	r=0.32	-		r = - 0.12	r = - 0.01	-	
Clinical attachment level	r=0.51	r=0.24	r=0.71	-	r = 0.06	r = 0.46	r = 0.62	-

Furthermore, a significant association was observed between probing depth and clinical attachment level in the control group. In contrast, within the test group, the clinical attachment level exhibited a statistically significant correlation with the gingival bleeding Index.

Analysis of hematological parameters

The mean serum levels of 1,25(OH)2D in the test group is 173.59 ± 52.60 and in the control group, it is 401.47 ± 99.81. A statistically significant distinction in 1,25(OH)2D levels was observed between the test and control groups (p = 0.0001) at a significance level of 5%. Additionally, the mean serum levels of TNF-α in the test group are 1078.09± 231.51, contrasting with 204.75±68.31 in the control group. There was a statistically significant difference between test and control groups with regards to TNF-α levels (p = 0.0001) at a 5% level of significance. The correlation between periodontal parameters (i.e., PI, GBI, PPD, and CAL) with hematological parameters, namely 1,25(OH)2D and TNF-α in both the test and control groups was evaluated using Karl Pearson’s correlation coefficient method. The analysis revealed no statistically significant associations as shown in Table 8.

**Table 8:** Correlation between periodontal parameters with hematological parameters in test and test groups by Karl Pearson’s correlation coefficient method. p value less than 0.05 is statistically significant SD: standard deviation SE: standard error

Hematological	Periodontal parameters	Control group			Test group		
		R-value	t-value	p-level	R-value	t-value	p-level
Serum TNF-α levels	Plaque index	0.05	0.32	0.74	-0.14	-0.78	0.44
	Gingival bleeding index	0.26	1.49	0.14	0.12	0.71	0.48
	Pocket depth	-0.03	-0.17	0.85	-0.05	-0.29	0.77
	Clinical attachment level	0.01	0.06	0.94	-0.20	-1.14	0.26
Serum 1,25(OH)_2_levels	Plaque index	-0.07	-0.39	0.69	-0.10	-0.56	0.57
	Gingival index	0.29	1.67	0.10	-0.20	-1.13	0.26
	Pocket depth	-0.22	-1.24	0.22	-0.08	-0.45	0.65
	Clinical attachment level	0.28	1.65	0.10	-0.10	-0.59	0.55

In accordance with the periodontal parameters, the serum 1,25(OH)2D level was notably higher in the control group than in the test group. This finding underscores a positive correlation between serum 1,25(OH)2D levels and periodontal health status.

## Discussion

The current diagnostic practices for periodontitis primarily rely on radiograph readings, clinical pictures, the patient’s medical and genetic history, and clinical observations. However, these methods only offer historical statistics on periodontal tissue damage. Serum levels of 25(OH)D, 1,25(OH)2D, ultrasensitive C-reactive protein, and high-density lipoprotein cholesterol were determined. To address this limitation, there is an urgent need for quick, efficient, and focused diagnostic and monitoring methods, including biochemical markers [12].

Vitamin D is one such marker due to its potential to influence attachment apparatus loss. Adequate vitamin D levels have been associated with a potential slowdown in disease progression by stimulating the production of cathelicidin. Human periodontal ligament cells and gingival fibroblasts have been found to activate the vitamin D pathway, thereby inducing the production of cathelicidin to support immunological response. Notably, serum 25(OH)D insufficiency has been linked to lower levels of human-defensin-2 and cathelicidin in gingivitis and chronic periodontitis, as indicated in select studies.

This study highlights statistically significant disparities between the recorded parameters of the test group and the control group. Correlation analyses reveal higher clinical parameters in the test group as compared to the control group. The data indicate a suggestive association where, in the test group, elevated periodontal parameters correspond to decreased 1,25(OH)2D serum levels and increased TNF-α serum levels. This statistically relevant difference corroborates the findings in the study by Gokul et al. [7] 

A study by Dietrich et al. on whether 25(OH)D3 serum concentrations are linked to the probing depth and/or attachment loss established that 25(OH)D3 serum concentrations were related inversely to periodontal attachment loss in patients aged above 50 but in those below 50 years, the linkage between 25(OH)D3 and attachment loss had been dismal [13].

No relation could be established between 25(OH)D3 and periodontal status in the studies conducted by Antonoglou et al. This discrepancy suggests that the low substrate availability for converting to 1,25(OH)2D might not be solely responsible for lower 1,25(OH)2D levels [14]. In the studies that followed, Antonoglou et al. administered anti-infection therapies to periodontitis patients, which included root planning, scaling, and similar oral hygiene treatments. Upon reassessing the clinical and laboratory parameters after two months, an increased concentration of 1,25(OH)2D in serum levels was observed [15]. This suggests a different explanation, that is, the patients diagnosed with low serum levels of 1,25(OH)2D may be prone to chronic periodontitis. A state when vitamin D deficiency may lead to the pro-inflammatory stage that is upregulated and thus causes an increase in TNF-α. In addition, factors such as bacterial burden, and virulence pointers such as lipopolysaccharide (LPS) may lead to an increase in TNF-α serum levels [16].

25(OH)D and 1,25(OH)2D were tested for their ability to inhibit the expression of interleukin-6 (IL-6), interleukin-8 (IL-8), and monocyte chemotactic protein-1 (MCP-1) by periodontal ligament cells after they were stimulated with Porphyromonas gingivalis LPS or heat-killed Porphyromonas gingivalis at 10-100 nm concentrations [15]. Similarly, Xu et al. found that 1,25(OH)2D prevented Porphyromonas gingivalis from boosting the synthesis of cytokines such as IL-6 while simultaneously increasing the expression of the anti-inflammatory cytokine IL-10 in macrophages. The phosphorylation of p38 mitogen-activated protein kinase (MAPK) and ERK1/2 was shown to be suppressed by 1,25(OH)2D in subsequent tests [17]. Moreover, Bashutski et al. assessed the impact of vitamin D supplementation with calcium on periodontal regeneration after surgical therapy in patients with severe chronic periodontal disease [17].

Limitations of the present study include its cross-sectional design and a small sample size due to which the temporal sequence between vitamin D levels and the outcome was difficult to establish. A causal analysis of vitamin D and periodontal diseases cannot be performed based on these cross-sectional studies.

Clinical relevance

Vitamin D supplementation can decrease the further or ongoing process of clinical attachment loss. Further randomized controlled and longitudinal studies with large sample sizes are needed to determine serum 1,25(OH)2D as a diagnostic marker for periodontal disease or a prognostic marker for determining the outcome of the disease outcome. Gingival crevicular fluid or tissue biopsy levels of 1,25(OH)2D should be warranted to prove its role in therapeutic modalities.

## Conclusions

Within the limitations of the study, the need for early-stage evaluation of biomarkers to detect periodontitis becomes evident. This study revealed a striking contrast: chronic periodontitis patients exhibited lower serum 1,25(OH)2D levels while concurrently displaying elevated serum TNF-α levels. Moreover, this study could reach some of the targets for evaluating the levels of 1,25-dihydroxycholecalciferol and TNF-α in a systematic way. It concludes that these biomarkers can serve as valuable tools for the early detection of chronic periodontitis. To further enrich our understanding, future investigations should encompass larger sample sizes and extended follow-up periods.
